# Kabuki syndrome: novel pathogenic variants, new phenotypes and review of literature

**DOI:** 10.1186/s13023-019-1219-x

**Published:** 2019-11-14

**Authors:** Huakun Shangguan, Chang Su, Qian Ouyang, Bingyan Cao, Jian Wang, Chunxiu Gong, Ruimin Chen

**Affiliations:** 1 Department of Endocrinology, Fuzhou Children’s Hospital of Fujian, Fujian Medical University Teaching Hospital, Fuzhou, 350000 China; 2 Department of Endocrinolgy, Genetics and Metabolism, Beijing Children’s Hospital, Capital Medical University, National Center for Children’s Health, Beijing, 100045 China; 3 Department of Molecular Genetic Diagnostics, Shanghai Children’s Medical Center, Shanghai Jiao Tong University School of Medicine, Shanghai, 200127 China

**Keywords:** Kabuki syndrome, KMT2D, KDM6A, Chinese patients, Brain abnormalities

## Abstract

**Objective:**

This study describes 5 novel variants of 7 *KMT2D/KDM6A* gene and summarizes the clinical manifestations and the mutational spectrum of 47 Chinese Kabuki syndrome (KS) patients.

**Methods:**

Blood samples were collected for whole-exome sequencing (WES) for 7 patients and their parents if available. Phenotypic and genotypic spectra of 40 previously published unrelated Chinese KS patients were summarized.

**Result:**

Genetic sequencing identified six *KMT2D* variants (c.3926delC, c.5845delC, c.6595delT, c.12630delG, c.16294C > T, and c.16442delG) and one *KDM6A* variant (c.2668-2671del). Of them, 4 variants (c.3926delC, c.5845delC, c.12630delG, and c.16442delG) in *KMT2D* gene and the variant (c.2668-2671del) in *KDM6A* gene were novel. Combining with previously published Chinese KS cases, the patients presented with five cardinal manifestations including facial dysmorphism, intellectual disability, growth retardation, fingertip pads and skeletal abnormalities. In addition, 29.5% (5/17) patients had brain abnormalities, such as hydrocephalus, cerebellar vermis dysplasia, thin pituitary and white matter myelination delay, corpus callosum hypoplasia and Dandy-Walker malformation.

**Conclusion:**

In this report, five novel variants in *KMT2D/KDM6A* genes are described. A subset of Chinese KS patients presented with brain abnormalities that were not previously reported. Our study expands the mutational and phenotypic spectra of KS.

## Introduction

Kabuki syndrome (KS, OMIM#147920) is a rare syndrome with multiple congenital anomalies. It was first reported by Japanese researchers Kuroki and Niikawa [1, 2]. KS is a heterogeneous condition, two causative genes having been identified so far. The causative gene of KS was identified in 2010 when Bögershausen et al. [3] reported de novo heterozygous variants in *KMT2D* gene, which is located on chromosome 12q13. Later, in 2012, variants in the *KDM6A* gene, which is located on chromosome Xp11.23, were identified as another causative gene for KS [4].

Consistent features of KS included distinctive facial dysmorphism (long palpebral fissures, depressed nasal tip and large ears), short stature, intellectual disability, skeletal abnormalities and dermatoglypic abnormalities. Other recurrent features such as congenital cardiac anomalies, ureter malformation and hip joint dislocation had been reported in non-Chinese KS patients [5]. In addition, uncommon features had also been reported. Topcu et al. reported perisylvian cortical dysplasia in a KS patient from Turkey [6]. However, there is little information about brain abnormalities in KS patients.

Herein, we analyzed 7 patients, and identified 7 deleterious *KMT2D/KDM6A* variants including 6 truncating and 1 missense variants. Of them, 5 variants were novel. To date, 40 sporadic Chinese KS patients had been reported [7–15]. We evaluated the phenotype spectra of all Chinese KS patients and paid particular attention to the brain abnormalities among a total of 47 unrelated Chinese KS patients.

## Subjects and methods

### Subjects

Seven patients with clinical presentation of Kabuki syndrome were enrolled from Fuzhou Children’s Hospital of Fujian and Beijing Children’s Hospital, China. This study was approved by the Ethics Committee of Fuzhou Children’s Hospital of Fujian, and written informed consents were obtained from the participants’ legal guardians.

### Whole-exome sequencing and variants interpretation

Genomic DNA was extracted from peripheral blood leukocytes of each patient. Blood samples from the parents were also collected if available. The whole-exome sequencing (WES) was performed at Shanghai Children’s Medical Center and MyGenostics, Beijing, China. An adaptor-ligated library was prepared using SureSelect Human All Exon Kit (Agilent Technologies, Santa Clara, America) according to the manufacturer’s protocol. Target regions were sequenced on an Illumina Hiseq X Ten System (Illumina, San Diego, America). Paired end reads were aligned to the GRCh37/hg19 human reference sequence. BAM files were generated by Picard and sequence variants were called by Genome Analysis Toolkit (GATK) Haplotype Caller.

Variants were annotated by TGex and putative pathogenic variants detected in the patients by WES were validated by Sanger sequencing. Variants were classified following the ACMG/AMP standards and guidelines [16].

## Results

### Clinical manifestations of seven Chinese patients with KS

We enrolled 7 patients with clinical diagnosis of KS (three males and four females). The age of initial diagnosis ranged from 7 days to 3.2 years. These patients exhibited a diverse phenotype. The clinical features of the seven Chinese patients are listed in Table 1. The main characteristics were as following: facial dysmorphism (*n* = 7), cardiac abnormalities (*n* = 6), intellectual disability (*n* = 5), short stature (*n* = 4), skeletal abnormalities (*n* = 3), hearing impairment (n = 3) and dermatoglypic abnormalities (*n* = 2).

**Table 1:** Phenotypic summary of Chinese KS patients

Patient	1	2	3	4	5	6	7	Literature (*N* = 40)	Chinese cohort (*N* = 47)	Non-Chinese cohort (*N* = 86) (Ref. 17)
Gender	Female	Female	Male	Female	Male	Male	Female			
Age of diagnosis	1.3 yrs	11 Months	5 Months	7d	7 yrs	2.6 Months	3.2 yrs			
Growth										
Short stature	+	–	–	–	+	+	+	23	57.4%	57%
Neurological abnormalities										
Intellectual disability	+	–	+	NA	+	+	+	32	80.4%	90%
Seizures	–	–	–	–	–	–	–	4	8.5%	15%
Cerebellar vermis dysplasia	–	–	–	–	–	–	–	1	2.1%	
Corpus callosum hypoplasia	–	–	–	–	–	–	–	1	2.1%	
Dany-Walker malformation	–	–	–	–	–	–	–	1	2.1%	
Thinning of pituitary	–	–	–	–	+	–	–	0	2.1%	
Delay myelination of cerebral	–	–	+	–	–	–	–	0	2.1%	
Hydrocephalus	–	–	–	–	–	–	–	1	2.1%	
Craniofacial features										
Microcephaly	–	+	+	–	–	–	–	3	10.6%	41%
Micrognathia	–	–	–	–	–	–	–	3	6.3%	39%
High forehead and hairline	+	–	–	–	–	–	–	0	2.1%	
Low hairline	+	–	–	–	–	–	–	2	6.3%	
Hypertelorism	–	–	+	–	–	+	–	8	21.2%	
Epicanthus	–	–	–	–	+	–	–	8	19.1%	
Long palpebral fissures	–	+	+	–	–	–	+	15	38.2%	99%
Strabismus	–	–	–	–	–	–	–	1	2.1%	37%
Eversion of lateral third of lower eyelids	+	–	+	–	+	–	+	14	38.2%	87%
Long eyelashes	+	–	–	–	–	–	+	9	23.9%	
Arched eyebrows	+	–	–	–	–	+	–	2	8.7%	
Sparse eyebrows	–	–	–	–	+	+	–	18	42.5%	
Depressed nasal tip	+	+	–	–	–	+	+	29	70.2%	80%
Wide nasal bridge	+	+	–	–	–	+	–	7	21.9%	
A displastic ear	–	+	–	–	–	–	–	3	8.7%	
Large ears	–	+	–	–	+	+	–	29	68.0%	79%
High-arched/cleft palate	–	+	+	–	–	–	+	24	57.4%	66%
Thin upper vermillion	+	–	–	–	+	–	+	2	10.6%	76%
Abnormal dentition	–	–	–	–	–	–	–	5	10.6%	51%
Congenital heart defect	+	+	+	+	–	+	+	14	42.6%	42%
Aortic coartation	–	+	–	–	–	–	–	1	4.3%	
Atrial septal defect	+	–	+	+	–	+	–	6	21.7%	
Ventricular septal defects	+	–	+	+	–	–	+	6	21.7%	
Patent ductus arteriosus	–	–	–	+	–	+	–	1	6.5%	
Patent foramen ovale	+	+	+	+	–	–	+	5	21.7%	
Aortic arch dysplasia	–	–	–	+	–	–	–	0	2.2%	
Internal organ problem										
Feeding difficulties	+	–	–	–	–	–	–	3	8.5%	
Anal atresia	–	–	–	–	–	+	–	3	8.5%	
Bilateral inguinal hernia	–	–	–	–	–	–	–	2	4.2%	
Splenomegaly	–	–	–	–	–	+	–	1	4.2%	
Cryptorchidism	–	–	–	–	–	–	–	1	2.%	
Hearing impairment	–	+	+	–	–	+	–	13	34.0%	25%
Otitis media	–	–	–	–	–	+	–	12	27.6%	
Cholesteatoma	–	–	–	–	+	–	–	2	6.4%	
Cochlear dysplasia	–	–	–	–	–	+	–	0	2.1%	
Renal/ureter malformation	–	–	–	+	+	+	–	2	10.6%	40%
Musculoskeletal features										
Hip joint dislocation	–	–	–	–	+	–	+	9	23.4%	26%
Right diaphyseal femoral fracture	–	–	–	–	–	–	+	0	2.1%	
Fifth finger clinodactyly	+	–	–	–	–	–	–	22	48.9%	84%
Absent palmer transverse crease	–	–	+	–	–	–	–	5	12.7%	
Fingertip pads	+	–	–	–	–	–	–	24	53.2%	89%
Endocrine										
Hypoglycemia	–	+	+	–	–	–	–	2	8.5%	7–8%
Early breast development		–	–	–	–	–	+	1	4.2%	28%

### Pathogenic variants in *KMT2D* and *KDM6A*

By WES, we identified six variants (c.3926delC/p.P1309Qfs*21, c.5845delC/p.Q1949Sfs*98, c.6595delT/p.Y2199Ifs*65, c.12630delG/p.Q4210fs*5, c.16294C > T/p.R5432W and c.16442delG/p.C5481Lfs*6) in exon 12, 27, 31, 39, 51 and 52 of *KMT2D* gene (NM_003482.3), respectively, and one variant (c.2668-2671del) in exon 18 of *KDM6A* gene (NM_021140.3). The variants identified (c.5845delC, c.2668-2671del and c.12630delG) in 3 patients were confirmed by Sanger sequencing, and they were absent from their parents. The other 4 patients’ parental DNA were not available for genetic testing. Four variants (c.3926delC, c.5845delC, c.12630delG and c.16442delG in *KMT2D* gene, and the variant in *KDM6A* gene) were novel. Those 6 frameshift variants were predicted to lead to nonsense-mediated decay of mRNA. These null variants can all be classified as pathogenic according to the ACMG/AMP standards and guidelines (c.3926delC, c.5845delC, c.6595delT, c.12630delG, c.16442delG and c.2668-2671del). The remaining missense variant c.16294C > T; p.R5432W in *KMT2D* gene has been previously reported [17]. The variant c.16294C > T; p.R5432W was predicted to be deleterious by multiple in silico software, including SIFT (damaging), PolyPhen-2 (probably damaging), MutationTaster (disease causing), PROVEAN (deleterious), and CADD (damaging). Therefore, it can be considered to be likely pathogenic.

### Phenotypic spectrum of 47 Chinese KS patients

Forty Chinese patients had been previously reported with *KMT2D/KDM6A* mutations. With the new 7 patients adding, we summarized the phenotypic features of a total of 47 Chinese KS patients (Table 1). The major clinical signs were as following: facial dysmorphisms (47/47; 100%), intellectual disability (36/45; 80%), short stature (27/47; 57.4%) patients, fingertip pads (25/47; 53.1%), finger clinodactyly (23/47; 48.9%), 5th finger clinodactyly (23/47; 48.9%), congenital cardiac anomalies (20/47; 42.5%) and hip joint dislocation (11/47; 23.4%). Additionally, brain imaging datasets were available for 17 patients and five patients (5/17, 29.4%) exhibited disparate brain anomalies.

## Discussion

The genotypic spectrum of 47 Chinese KS patients (23 females, 24 males, 3 are sibs), including 42 *KMT2D* variants and 3 *KDM6A* variants were summarized (Table 2). Of the 42 *KMT2D* variants, there are 1 splicing, 1 non-frameshift indel, 10 nonsense, 13 frameshift and 17 missense variants. All of the nonsense and frameshift variants were categorized as pathogenic because the protein structure was significantly altered. We used silico prediction models including PolyPhen-2, PROVEAN, MutationTaster to analyze the missense variants. Two missense variants (c.7130C > T and c.11638C > A) are predicted to be benign, neutral or polymorphism by at least two of the three silico prediction models. The pathogenicity of the two variants (c.7130C > T and c.11638C > A) was inconclusive and could potentially be non-pathogenic according ACMG/AMP standards and guidelines. The p.R5432W variant was most common, observed in 3 unrelated patients (P2, P28 and P46), which may be a hot spot for *KMT2D* gene variation in Chinese Patients. Thirty four *KMT2D* variants and 3 *KDM6A* variants were confirmed by Sanger sequencing. Of them, 2 variants (c.16273C > A and c.7130 C > T) in *KMT2D* gene were inherited from their respective biological father, and 1 variant (c.335-1G > T) in *KDM6A* were inherited from mother, whereas the other 34 variants were de novo.

**Table 2:** Genotypic summary of Chinese KS patients

Case ID	Literature	Genes involve	Mutation	Preticted protein changes	Type of mutation	Inheritance	Exon	Pathogenic classification
1	This study	*KMT2D*	c.5845delC	p.Q1949Sfs*98	Frameshift del	De novo	27	Pathogenic
2		*KMT2D*	c.16294C > T	p.R5432W	Missense	NA	51	Likely Pathogenic
3		*KDM6A*	c.2668-2671del	p.N891Vfs*27	Frameshift del	De novo	18	Pathogenic
4		*KMT2D*	c.6595delT	p.Y2199Ifs*65	Frameshift del	NA	31	Pathogenic
5		*KMT2D*	c.16442delG	p.C5481Lfs*6	Frameshift del	NA	52	Pathogenic
6		*KMT2D*	c.3926delC	p.P1309Qfs*21	Frameshift del	NA	12	Pathogenic
7		*KMT2D*	c.12630delG	p.Q4210fs*5	Frameshift del	De novo	39	Pathogenic
8	[7] Liu S, et al. BMC Med Genet. 2015, 16:26.	*KMT2D*	c.12199C > T	p.P4067Sr	Missense	De novo	39	Likely Pathogenic
			c.16295G > A	p.R5432Q	Missense	De novo	51	Likely Pathogenic
9		*KMT2D*	c.4664C > T	p.S1555F	Missense	De novo	17	Likely Pathogenic
10		*KMT2D*	c.8639 T > C	p.L2880P	Missense	De novo	34	Likely Pathogenic
11		*KMT2D*	c.3095delT	p.L1032Rfs24X	Frameshift del	NA	11	Pathogenic
12		*KMT2D*	c.96C > G	p.D32E	Missense	De novo	2	Likely Pathogenic
13		*KMT2D*	c.4395dupC	p.K1466Qfs25X	Frameshift del	NA	15	Pathogenic
14		*KMT2D*	c.11638C > A^a^	p.L3880 M	Missense	NA	39	Uncertain significance
*15*		*KMT2D*	c.4140 T > A	p.C1370X	Nonsense	NA	14	Pathogenic
			c.11718-11723delGCAACA		Non-Frameshift indel	NA	39	Likely Pathogenic
16	[8] Yang P, et al. Am J Med Genet A. 2016, 170 (6): 1613–21.	*KDM6A*	exon1-2del		Frameshift del	De novo		Pathogenic
17	[9] Wu BB, et al. Chin J Evid Based Pediatr. 2017, 12 (2):135–9.	*KMT2D*	c.12697C > T	p.Q4233X	Nonsense	De novo	39	Pathogenic
			c.12696C > T	p.Q4232H	Missense	De novo	39	Pathogenic
18		*KMT2D*	c.3495delC	p.P1165Lfs*47	Frameshift del	De novo	11	Pathogenic
*19*		*KMT2D*	c.10881delT	p.L3627Rfs*31	Frameshift del	De novo	39	Pathogenic
*20*		*KMT2D*	c.16498C > T	p.R5500W	Missense	NA	53	Likely Pathogenic
*21*		*KMT2D*	c.12560G > A	p.G4187E	Missense	NA	39	Likely Pathogenic
*22*		*KMT2D*	c.16273G > A	p.E5425K	Missense	NA	51	Likely Pathogenic
*23*	[10] JUN LU, et al. MOLECULAR MEDICINE REPORTS. 2016, 14: 3641–3645.	*KMT2D*	c.4485C > A	p.Y1495S	Missense	De novo	16	Pathogenic
*24*	[11] Chengqi Xin, BMC Medical Genetics. 2018, 19:31	*KMT2D*	c.5235delA	p.A1746Lfs*39	Frameshift del	De novo	22	Pathogenic
*25*		*KMT2D*	c.7048G > A	p.Q2350*	Frameshift del	De novo	31	Pathogenic
*26*	[12] Ju-Li Lin, et al. Clinical Genetics, 2015, 88 (3): 255–260.	*KMT2D*	c.12307C > T	p.Q4013X	Nonsense	De novo	38	Pathogenic
*27*		*KMT2D*	c.3754C > T	p.R1252X	Nonsense	De novo	11	Pathogenic
*28*		*KMT2D*	c.16294C > T	p.R5432W	Nonsense	De novo	51	Likely Pathogenic
*29*		*KMT2D*	c.5993A > G	p.Y1998C	Missense	De novo	28	Likely Pathogenic
*30*		*KMT2D*	c.16273G > A	p. E5425K	Missense	*Father*	51	Likely Pathogenic
*31*		*KMT2D*	c.16273G > A	p. E5425K	Missense	*Father*	51	Likely Pathogenic
*32*		*KMT2D*	c.16273G > A	p. E5425K	Missense	*Father*	51	Likely Pathogenic
*33*		*KMT2D*	c.8743C > T	p.R2915X	Nonsense	De novo	34	Pathogenic
*34*		*KMT2D*	c.5269C > T	p.R1757X	Nonsense	De novo	22	Pathogenic
*35*		*KMT2D*	c.16273G > A	p.E5425K	Missense	De novo	51	Likely Pathogenic
*36*		*KMT2D*	c.7650-1delCT	p.P2550Rfs2604X	Frameshift del	De novo	31	Pathogenic
*37*		*KMT2D*	c.16135C > T	p.Q5379X	Nonsense	De novo	51	Pathogenic
*38*		*KMT2D*	c.15326G > T	p.C5109F	Missense	De novo	48	Pathogenic
*39*		*KMT2D*	c.16498C > T	p.R5500W	Missense	De novo	53	Pathogenic
*40*	[13] LI Jieling, ea. al. J Clin Pediatr. 2018, 1 (36): 53–56.	*KMT2D*	c.7130C > T^a^	p.P2377L	Missense	Father	31	Uncertain significance
*41*		*KMT2D*	IVS9 + 2 T > G		Splice mutation	De novo		Pathogenic
*42*	[14] Wang Hongmei, et al. Chin J Pediatr. 2018, 56 (11): 846–849.	*KMT2D*	c.11770C > T	p.Q3924X	Nonsense	De novo	39	Pathogenic
*43*		*KMT2D*	c.13033A > T	p.K4345X	Nonsense	De novo	39	Pathogenic
*44*		*KMT2D*	c.1763C > G	p.S588X	Nonsense	De novo	10	Pathogenic
*45*		*KMT2D*	c.5848delT	p.S1950Pfs*97	Frameshift	De novo	27	Pathogenic
*46*		*KMT2D*	c.16294C > T	p.R5432W	Missense	De novo	51	Likely Pathogenic
*47*	[15] Guo Z,et al. BMC Med. Genet. 2018, 12 03;19 (1).	*KDM6A*	c.335-1G > T		Splice site mutation	*mother*		Likely Pathogenic

^a^No sufficient evidence supporting it’s pathogenicity *Denotes a frameshift change as the first affected amino acid

A phenotypic comparison between the 47 Chinese patients and a cohort of 86 patients from other populations was showed in Table 1. It was reported that the long palpebral fissures were observed in 99% of non-Chinese KS patients, and the eversion of lateral third of lower eyelids 87% [17]. The Chinese patients showed a significantly lower frequency (38.2% for both features). While a lack of clinical acuity in recognizing these features by clinicians could account for some differences, we think it may more likely reflecting the ethnicity difference in feature presentations. Additionally, The Chinese patients had higher frequency of hearing impairment but lower frequency of microcephaly, micrognathia, strabismus, abnormal dentition, fifth finger clinodactyly and fingertip pads. The frequencies of other phenotypes including short stature, intellectual disability, cardiac defects, large eras, hypoglycemia and high-arched/cleft palate were consistent with previously reported [17].

*KMT2D/KDM6A* affects genes and biological processes globally. The clinical consequence of *KMT2D/KDM6A* gene mutations also seems to have a global effect on development and growth, both craniofacial, cardiac, neural and musculoskeletal (presented with short stature) tissue [18]. Across the board, the Chinese KS patients had typical facial features. These dysmorphic features included long palpebral fissures, depressed nasal tip and large ears (most prominent from the profile), similar to the KS patients from other ethnicities, indicating a consistent and highly penetrant facial dysphormic profile across populations.

Thirty-one Chinese patients presented with intellectual disability, most were mildly affected. Mehmet et al. [19] reported one and Parisi et al. [20] reported three KS patients with autism spectrum disorder, yet none of the Chinese KS patients exhibited autistic features or significant behavioral issues. Various structural brain anomalies had been infrequently described in KS patients. Topcu et al. reported perisylvian cortical dysplasia [6]. Cedrik et al. reported two patients presented with holoprosencephaly [21]. Furthermore, based on MRI, significantly decreased grey matter volume in the bilateral hippocampus and dentate gyrus have been described in KS patients [22]. We found the brain abnormalities including thinning of pituitary and myelination of cerebral white matter in Chinese KS patients, which were not previously reported in KS patients. We also found that hydrocephalus, corpus callosum hypoplasia and Dandy-Walker malformation which had been reported previously both in Chinese patients and other populations [7, 15]. In addition, cerebellar vermis dysplasia was initially reported in Chinese patients [11]. These observations suggested a strong association between various brain abnormalities and KS. Further study is needed to explore the clinical consequences of these brain abnormalities.

## Conclusions

We described five novel variants that are causal for the seven KS Chinese patients, and confirmed that the Chinese KS presented with typical clinical phenotypes as previously reported in non-Chinese patients, but of variable feature prevalence. We also pointed out that brain structural abnormalities including thinning of pituitary and delay myelination of cerebral white matter may be part of KS phenotype that warrant further investigation.

## Data Availability

The dataset supporting the conclusions of this article is included within the article.

## Abbreviations

ACMG/AMP: American College of Medical Genetics and Genomics and the Association for Molecular Pathology
*KDM6A*: lysine (K)-specific methylase 6A
*KMT2D*: lysine (K)-specific methyltransferase 2D
KS: Kabuki syndrome
WES: whole-exome sequencing
